# The Effects of Sesamin Supplementation on Obesity, Blood Pressure, and Lipid Profile: A Systematic Review and Meta-Analysis of Randomized Controlled Trials

**DOI:** 10.3389/fendo.2022.842152

**Published:** 2022-03-04

**Authors:** Yiting Sun, Jingyi Ren, Siqi Zhu, Zhenao Zhang, Zihao Guo, Jiaqi An, Bowen Yin, Yuxia Ma

**Affiliations:** ^1^ Undergraduate of College of Basic Medicine, Hebei Medical University, Shijiazhuang, China; ^2^ Department of Nutrition and Food Hygiene, School of Public Health, Hebei Medical University, Hebei Province Key Laboratory of Environment and Human Health, Shijiazhuang, China; ^3^ Undergraduate of College of Public Health, Hebei Medical University, Shijiazhuang, China

**Keywords:** sesamin, obesity, blood pressure, lipid profile, meta-analysis

## Abstract

**Aims:**

Sesamin, the main lignin constituent of sesame, plays a pivotal role in regulating physical state. Some studies have evidenced that the supplementation of sesamin may decrease cardiovascular disease risk. The goal of this systematic review was to summarize evidence of the effects of sesamin supplementation on obesity, blood pressure, and lipid profile in humans by performing a meta-analysis of randomized controlled trials.

**Data Synthesis:**

Five databases (PubMed, Cochrane Library, EMBASE, Web of Science, and Scopus) were searched electronically from inception to July 2021 to identify randomized controlled trials that assessed the impact of sesamin on obesity, blood pressure, and lipid profile. Weighted mean difference (WMD) and standard deviation (SD) were used to present the major outcomes.

**Conclusions:**

Seven trials (n = 212 participants) were included in the overall analysis. Results showed that sesamin supplementation caused a great reduction in TC (WMD: -10.893 mg/dl, 95% CI: −19.745 to −2.041, p = 0.016), LDL-c (WMD: -8.429 mg/dl, 95% CI: −16.086 to −0.771, p = 0.031), and SBP (WMD: −3.662 mmHg, 95% CI: −6.220 to −1.105, p = 0.005), whereas it had no effect on HDL-c, TG, DBP, or weight. Subgroup analysis showed that duration, parallel design, and unhealthy status can affect TC, LDL-c, and SBP evidently. We did not discover a strong link between indicators’ changes and duration of supplementation. Sesamin can be used as an obtainable dietary supplement to improve blood pressure and blood lipids, and further as a health product to prevent cardiovascular diseases.

## 1 Introduction

At the global level, the highest per capita cardiovascular disease (CVD) burden remains in the countries of Eastern Europe and Central Asia (1). Several published articles have mentioned that potentially modifiable risk factors, such as high blood pressure (BP), raised serum lipids, and obesity may play key roles in promoting the pathogenesis of CVDs (2–4). Blood profile levels are good indicators of cardiovascular risk and good predictors of coronary disease outcome (5). Dyslipidemia, defined as elevated levels of triglycerides and cholesterol (particularly LDL-c) and reduced levels of HDL-c, has been introduced as a strong risk factor for CVD (6, 7). High blood lipid levels can result in serious damage to systemic blood vessels and organs (8, 9). Several significant associations have been shown between CVD burden and circulati1ng levels of LDL-c, HDL-c, and triglycerides (10, 11). Hypertension (systolic blood pressure ≥140 mmHg or diastolic blood pressure ≥90 mmHg) (12) and CVDs are inseparable, too.

Sesamin, which constitutes 1.5% of the weight of sesame seed (13), has been consumed as a health natural supplement. This nutrient is also present in several plants distributed in different genera, including camellia, magnolia, piper, sesamum, and virola (14). It has traditionally been believed to have health benefits in some East Asian countries for many years. In the recent decades, it has been shown that sesamin exhibits several physiological actions in animals, such as antiobesity, antihypertensive, and serum lipid–lowering effects (15–17). The animal experiment showed that sesamin has been used to decrease blood lipids and blood glucose levels in the aorta of rats with metabolic syndrome (18). Several animal studies have also confirmed that the supplementation of sesame seeds or sesamin could decrease cholesterol levels (19, 20).

Hirata et al. (21) have experimented with sesamin on human subjects, and the results were surprising. TC and LDL-c were significantly lower in the sesamin-treated group. Similarly, the results obtained by Mohammadshahi (22) also had an effect on TC and LDL-c. Up to now, randomized controlled trials (RCTs) have not reached a consistent conclusion about the effect of sesamin on blood pressure and lipid profile (23, 24).

Given the evidence that sesamin is related to a decreased risk of CVD, we carried out a systematic review and meta-analysis, which aims at determining whether sesamin intake has the potential to be used as an adjuvant therapy for persons who have cardiovascular disease.

## 2 Materials and Methods

This study was executed based on the Preferred Reporting Items for Systematic Reviews and Meta-Analyses (PRISMA) guidelines (25) and registered in the International Prospective Register of Systematic Reviews (PROSPERO) database under the registration number CRD42021271145.

### 2.1 Search Strategy

Systematic literature retrieval was performed in the PubMed, SCOPUS, Cochrane Library, Embase, and ISI Web of Science databases from inception to July 2021 to determine a randomized controlled trial evaluating the effects of sesamin on obesity, blood pressure, and lipid profile. Medical subject heading terms (Mesh) were used: (“sesame” OR “sesamin” OR “sesamum”) AND (“Blood Pressure” OR “Hypertension” OR “High Blood Pressure”) AND(”HDL” OR “LDL” OR “Triglyceride” OR “Total cholesterol”) AND (“BMI” OR “Weight”) (**Supplementary Material**, which illustrates the search strategies). Then, the retrieved manuscripts were imported into EndNote software (version X9) to remove the duplicates. The inclusion and exclusion criteria are listed in **Table 1**. Two authors (YS and JR) independently and cooperatively determined suitable manuscripts for inclusion. Disagreements were discussed by the third author (SZ).

**Table 1:** PICOS criteria for inclusion of studies.

Parameter	Description
Population	Adult participants (healthy/unhealthy)
Intervention	I Sesamin administered for ≥2 weeks
	II Sesamin dosage is clearly indicated
Comparator	Placebo
Outcomes	Outcomes regarding at least one of the following markers: cholesterol, total cholesterol, high-density lipoprotein, low-density lipoprotein, triglycerides, triacylglycerol, VLDL, BMI, weight, blood pressure, diastolic blood pressure, systolic blood pressure
Study design	Randomized placebo-controlled clinical trial with a cross-over or parallel design

### 2.2 Data Extraction

Based on the pre-designed table, the important report data are listed as the following: publication information (first author’s last name, the year published, study location), the details of the clinical trial (study design, intervention duration), the participants’ characteristics (sample size, age, gender, health status), and all reported outcomes of interest. Standard deviation (SD), belonging to the category of descriptive statistics, was the experimental index to be captured. When SE was reported, we use the formula between SD and SEM (SD = SEM × sqrt (n); n = number of participants) to convert.

### 2.3 Assessment of Quality

Trials were assessed for bias risk using the Cochrane Bias Risk Tool (26) which includes sequence generation, allocation concealment, blinding, blinding of outcome assessment, incomplete outcome data, selective outcome reporting, and other bias. We ranked for “low”, “high”, or “unclear” risk of bias.

### 2.4 Quantitative Data Synthesis

All the analyses were performed using STATA version 11. Weighted mean difference (WMD), SD, and 95% CI were used as the effective measures for SBP, DBP, HDL-c, LDL-c, TG, TC, and weight. The net changes in them were equal to the post-intervention values minus the baseline values. The SD of the mean difference was calculated by the following formula:


$$\text{SD}={\text{square root [(SD}}_{\text{pre}-\text{treatment}}{)}^{2}{\text{+(SD}}_{\text{post}-\text{treatment}}{)}^{2}-(2\text{R}\times {\text{SD}}_{\text{pre}-\text{treatmen}}\times {\text{SD}}_{\text{post}-\text{treatment}})]$$

assuming a correlation coefficient (R) = 0.5 for both the pre-test/post-test (parallel groups) and the crossover designed studies. The heterogeneity index I^2^ is used for quantitative analysis of heterogeneity, which ranges from 0% to 100%. There is no heterogeneity at 0%. The greater the I^2^ value, the greater the heterogeneity. There was no statistical heterogeneity (I^2^ < 50%) among the results of each study, and a fixed-effect model was used. If there was statistical heterogeneity (I^2^ ≥ 50%) among the results, the random-effect model was used.

### 2.5 Meta-Regression Analysis

Meta-regression analysis was performed to calculate the duration–effect relationship between WMD and duration to explore potential explanations for heterogeneity.

### 2.6 Subgroup Analysis

We also conducted subgroup analysis studies including treatment duration (<42 days or ≥42 days), study design (crossover or parallel), and participants’ health status (healthy or unhealthy). To evaluate the influence of individual study on the pooled-effect size, sensitivity analysis (leave-one-out) was conducted and p < 0.05 was considered as statistically significant.

### 2.7 Publication Bias

Begg’s rank correlation and Egger’s weighted regression statistics were used to evaluate potential publication bias (27, 28). p values less than 0.1 were considered statistically significant.

## 3 Results

### 3.1 Flow and Characteristics of the Included Study

**Figure 1:**
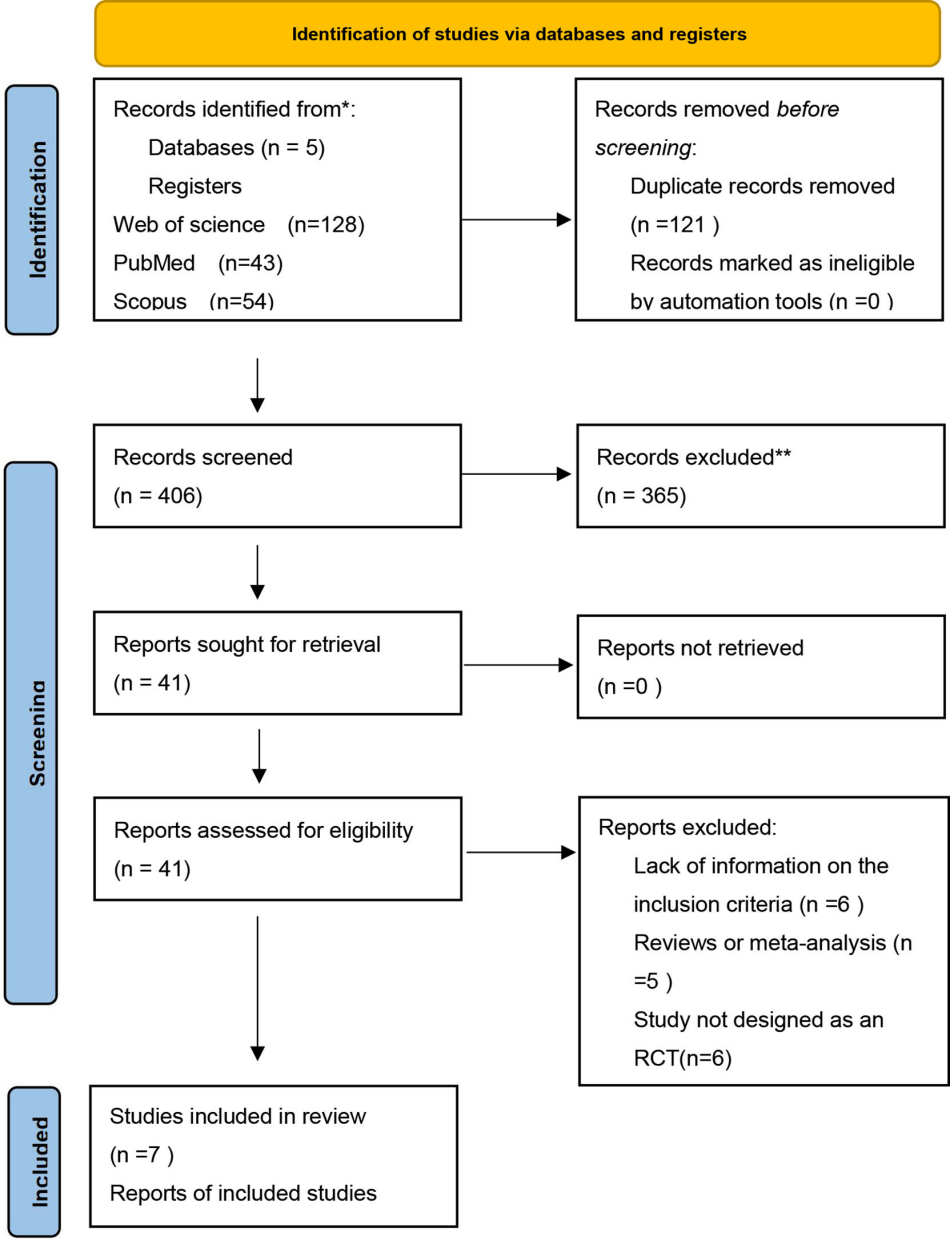
The PRISMA flowchart.

The initial search identified 527 papers for screening, of which 121 were removed because of duplication (**Figure 1**). After title and abstract screening, 365 records were excluded due to irrelevance to the inclusion criteria. The full texts of the remaining 41 articles were further screened, after which 34 studies were excluded for the following reasons: lack of sufficient information on the outcomes of interest (n = 6); the dosage of sesamin was not specified (n = 17); study not designed as an RCT (n = 6); and article published as meta-analysis or review (n = 5). Finally, 7 articles (15, 21–24, 29, 30) with 212 arms were enrolled in the present meta-analysis. The PRISMA flowchart of the study is shown in the following.

### 3.2 Characteristics and Quality of Included Studies

Characteristics of the included studies are shown in **Table 2**. Each of the included articles stated sesamin dosage, and four of the studies had dosages less than 200 mg/day. Four studies were on obesity, four studies were on BP, and five studies were on lipid profile. Included studies have been published between 1996 and 2016 and were conducted in 5 different areas: Japan, Taiwan, Australia, Thailand, and Iran. A total of 212 participants were enrolled in studies, and intervention duration ranged from 28 to 60 days. Four studies had a parallel design and three studies had a crossover design. Six trials were conducted in unhealthy individuals, and one trial was carried through healthy individuals.

**Table 2:** Characteristics of study populations, type of interventions, and study designs in the included trials.

Reference	Study design	Journal	Country/area	Sample size	Sex (M/F)	Target population	Mean age (y)	BMI (kg/m^2^)	Intervention/control	Duration	Main outcomes	Notes
Hirata (21)	R,PC,P	Atherosclerosis	Japan	12	12/0	Hypercholesterolemia	ns	ns	Placebo/sesamin	8 weeks	TC TG LDL-c HDL-c	Mean SD
Wu (29)	R,PC,C	American Society for Nutrition	Taiwan	24	0/24	Healthy postmenopausal women	59	18-28	Placebo/sesame	5 weeks	Weight TC VLDL-c LDL-c HDL-c TG VLDL-TG	Mean SD
Miyawaki (15)	R,DB,PC,C	J Nutr Sci Vitaminol	Japan	25	23/2	Mild hypertension	49.1	24.6	Placebo/sesamin	4 weeks	SDP BDP BMI	Mean SE
Wu (23)	R,PC,C	Elsevier	Australia	33	18/15	Overweight men and post-menopausal women	54.7	30.8	Placebo/sesame	5 weeks	Weight HDL-c LDL-c TC SBP DBP	Mean SD
Wichitsranoi (24)	R,PC,P	Nutrition Journal	Thailand	30	8/22	With prehypertension	49.8	26.1	Placebo/sesame	4 weeks	SDP BDP	Mean SD
Helli (30)	R,DB,PC,P	Journal of the American College of Nutrition	Iran	44	0/44	Rheumatoid arthritis	55.49	32.8	Placebo/sesamin	6 weeks	Weight BMI SBP DBP TG TC HDL-c LDL-c	Mean SD
Mohammadshahi (22)	R,DB,PC,P	J Babol Univ Med Sci	Iran	44	22/22	With type II diabetes	50.86	29.15	Placebo/sesamin	60 days	Weight BMI total TC LDL-c HDL-c	Mean SD

C, crossover; CVD, cardiovascular disease; DB, double-blind; DBP, diastolic blood pressure; HDL-c, high-density lipoprotein cholesterol; LDL-c, low-density lipoprotein cholesterol; P, parallel; PC, placebo controlled; R, randomized; RA, rheumatoid arthritis; SBP, systolic blood pressure; TC, total cholesterol; TG, triglycerides.

### 3.3 Findings From Meta-Analysis

**Figure 2:**
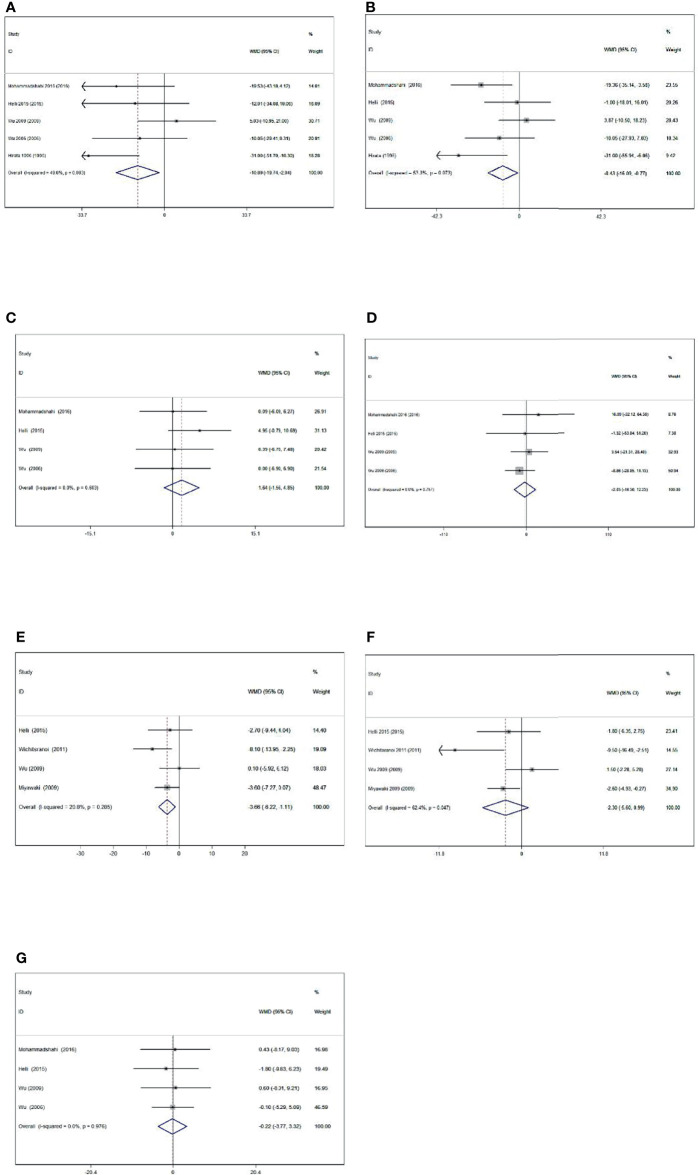
Forest plot of the effect of sesamin supplementation on TC **(A)**, LDL-c **(B)**, HDL-c **(C)**, TG **(D)**, SBP **(E)**, DBP **(F)**, and weight **(G)**.

Meta-analysis showed that sesamin supplementation caused a great reduction in TC (WMD: -10.893 mg/dl, 95% CI: −19.745 to −2.041, p = 0.016), LDL-c (WMD: -8.429 mg/dl, 95% CI: −16.086 to −0.771, p = 0.031), and SBP (WMD: −3.662 mmHg, 95% CI: −6.220 to −1.105, p = 0.005) (**Figure 2**).

#### 3.3.1 Effect of Sesamin Supplementation on Obesity Levels

Levels of obesity were reported in four of the included studies, including 145 participants. Sesamin ingestion showed a non-significant effect on mean body weight (WMD: -0.223, 95% CI: -3.766 to 3.321; p = 0.902) compared with control, without heterogeneity among the studies (I^2^ = 0.0%, p = 0.976).

#### 3.3.2 Effect of Sesamin Supplementation on BP Levels

The effect of sesamin on BP was reported in four treatments with 132 participants. Sesamin intake did significantly affect SBP (WMD: -3.662 mmHg, 95% CI: -6.220 to -1.105, p = 0.005; I^2^ = 20.8%). However, the pooled result using a random-effect model showed a reduction in DBP (WMD = -2.304 mmHg, 95% CI: -5.596 to 0.988, p = 0.170) with sesamin intake. A high heterogeneity was also detected in DBP (I^2^ = 62.4%).

#### 3.3.3 Effect of Sesamin Supplementation on Lipid Profile Levels

Five trials with 157 participants measured the effect of sesamin supplementation on TC and LDL-c. Moreover, the results of TC (WMD = -10.893 mg/dl, 95% CI: -19.745 to -2.041, p = 0.016; I^2^ = 49.8%) and LDL-c (WMD = -8.429 mg/dl, 95% CI: -16.086 to -0.771, p = 0.031; I^2^ = 53.3%) are detected following sesamin supplementation. With random-effect models, the I^2^ value of LDL-c was 53.3%, and the related p value was 0.073.

Four trials with 145 participants consuming sesamin affected HDL-c and TG. HDL-c (WMD = 1.644 mg/dl, 95% CI: -1.560 to 4.848, p = 0.314; I^2^ = 0.0%) and TG (WMD = -2.034 mg/dl, 95% CI: -16.298 to 12.229, p = 0.780; I^2^ = 0%) concentrations did not alter significantly following sesamin intake.

### 3.4 Risk-of-Bias Assessment

The quality of studies was evaluated by using the Cochrane collaboration’s risk-of-bias assessment tool. Random sequence generation, allocation concealment, and blinding of outcome assessment of participants were low risk of bias in all included studies. Only one trial had a high risk of bias due to the incomplete outcome. Details of the quality of studies are shown in **Table 3**.

**Table 3:** Assessment of risk of bias in studies included in the meta-analysis.

	Random sequence generation	Allocation concealment	Blinding of participants	Blinding of outcome assessment	Free of incomplete outcome	Free of selective reporting	Other bias
Hirata (21)	L	L	U	L	H	L	U
Wu (29)	L	L	U	L	L	L	L
Miyawaki (15)	L	L	L	L	L	L	U
Wu (23)	L	L	L	L	U	L	U
Wichitsranoi (24)	L	L	L	L	L	L	U
Helli (30)	L	L	L	L	L	U	L
Mohammadshahi (22)	L	L	L	L	L	L	L

L, low risk of bias; H, high risk of bias; U, unclear risk of bias.

### 3.5 Meta-Regression Analysis

**Figure 3:**
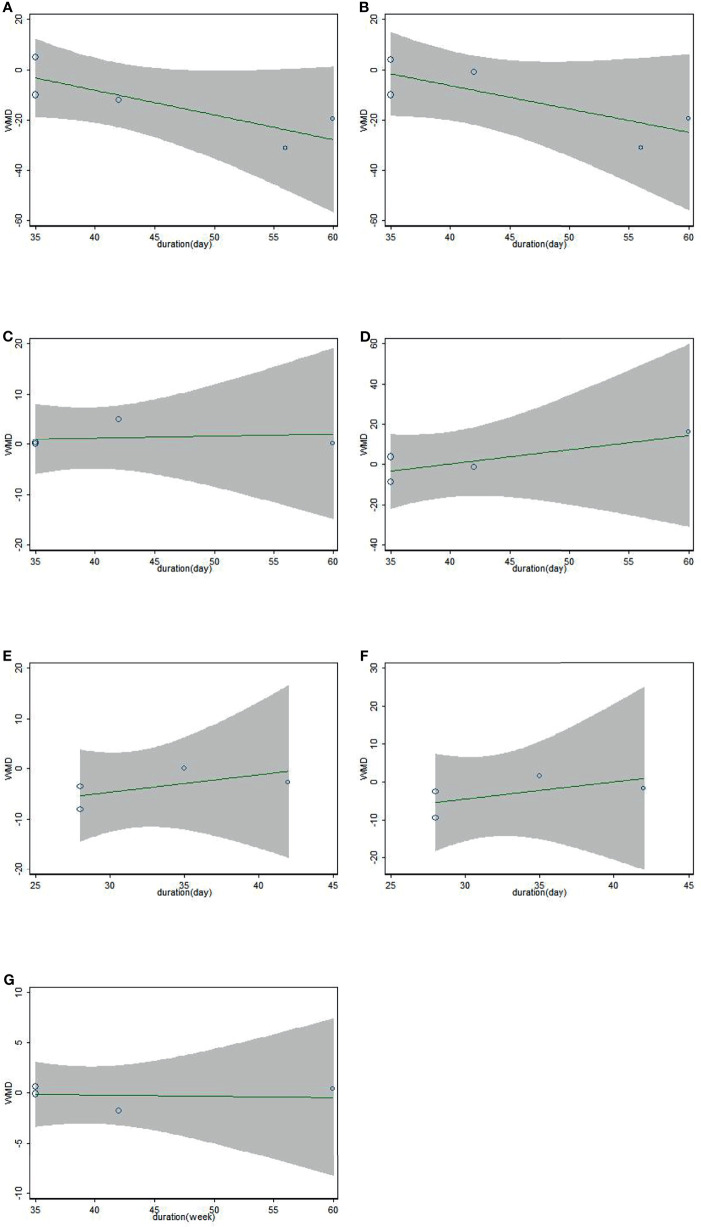
Association between duration of study and effect size of sesamin supplementation on TC **(A)**, LDL-c **(B)**, HDL-c **(C)**, TG **(D)**, SBP **(E)**, DBP **(F)**, and weight **(G)**.

Results of meta-regression suggested no clear relationship between the duration and biomarkers we conducted (TC: coefficient = 0.377, p = 0.671; LDL-c: coefficient = 0.395, p = 0.686; HDL-c: coefficient = 1.043, p = 0.988; TG: coefficient = 2.039, p = 0.806; SBP: coefficient = 1.414, p = 0.921; DBP: coefficient = 1.575, p = 0.897; weight: coefficient = 0.987, p = 0.996; **Figure 3**).

### 3.6 Subgroup Analysis

Subgroup analyses showed no significant differences in the effect of sesamin on HDL-c, TG concentrations, and weight between subgroups, which are stratified by intervention duration (≥42 days vs.<42 days), study design (parallel vs. crossover), and health status (healthy vs. unhealthy) (**Table 4**). It indicated that reduction was greater in trials conducted with longer duration, as for TC (WMD = -21.363 mg/dl, 95% CI: -34.090 to -8.636, p = 0.001; I^2^ = 0.0%) and LDL-c (WMD = -14.434 mg/dl, 95% CI: -24.929 to -3.939, p = 0.007; I^2^ = 55.2%). However, the reduction was more remarkable in participants with shorter duration (<42 days), as for SBP (WMD = -3.824 mmHg, 95% CI: -6.588 to -1.060, p = 0.007; I^2^ = 45.9%). In addition, the trials conducted in unhealthy participants showed a remarkable reduction in TC (WMD = -21.363 mg/dl, 95% CI: -34.09 to -8.636, p = 0.001; I^2^ = 0.0%), LDL-c (WMD = -14.434 mg/dl, 95% CI: -24.929 to -3.939, p = 0.007; I^2^ = 55.2%), SBP (WMD = -4.490 mmHg, 95% CI: -7.315 to -1.666, p = 0.002; I^2^ = 0.0%), and DBP (WMD = -3.542 mmHg, 95% CI: -6.873 to -0.210, p = 0.037; I^2^ = 46.0%). Subgroup analysis also suggested that trials with a parallel design were correlated with its reduction of TC (WMD = -21.363 mg/dl, 95% CI: -34.090 to -8.636, p = 0.001; I^2^ = 0.0%), LDL-c (WMD = -14.434 mg/dl, 95% CI: -24.929 to -3.939, p = 0.007; I^2^ = 55.2%), and SBP (WMD = -5.778 mmHg, 95% CI: -10.197 to -1.360, p = 0.010; I^2^ = 28.9%).

**Table 4:** Subgroup analyses.

Variables^2^	Duration		Health status		Study design	
	≥42 days	<42 days	Healthy	Unhealthy	Parallel	Crossover
***TC***						
No. of treatment arms	3	2	2	3	3	2
WMD (95% CI)	-21.363 (-34.090 to -8.636)	-1.082 (-13.402 to 11.238)	-1.082 (-13.402 to 11.238)	-21.363 (-34.090 to -8.636)	-21.363 (-34.090 to -8.636)	-1.082 (-13.402 to 11.238)
I^2^ (%)	0	27.9	27.9	0	0	27.9
*P*	0.001	0.863	0.863	0.001	0.001	0.863
***LDL-C***						
No. of treatment arms	3	2	2	3	3	2
WMD (95% CI)	-14.434 (-24.929 to -3.939)	-1.593 (-12.791 to 9.605)	-1.593 (-12.791 to 9.605)	-14.434 (-24.929 to -3.939)	-14.434 (-24.929 to -3.939 )	-1.593 (-12.791 to 9.605)
I^2^ (%)	55.2	29.3	29.3	55.2	55.2	29.3
*P*	0.007	0.780	0.780	0.007	0.007	0.780
***HDL-C***						
No. of treatment arms	2	2	2	2	2	2
WMD (95% CI)	2.697 (-1.509 to 6.902)	0.188 (-4.758 to 5.135)	0.188 (-4.758 to 5.135)	2.697 (-1.509 to 6.902)	2.697 (-1.509 to 6.902)	0.188 (-4.758 to 5.135)
I^2^ (%)	21.6	0	0	21.6	21.6	0
*P*	0.209	0.941	0.941	0.209	0.209	0.941
***TG***						
No. of treatment arms	2	2	2	2	2	2
WMD (95% CI)	8.129 (-27.385 to 43.644)	-3.989 (-19.564 to 11.586)	-3.989 (-19.564 to 11.586)	8.129 (-27.385 to 43.644)	8.129 (-27.385 to 43.644)	-3.989 (-19.564 to 11.586)
I^2^ (%)	0	0	0	0	0	0
*P*	0.654	0.616	0.616	0.654	0.654	0.616
***SBP***						
No. of treatment arms	1	3	1	3	2	2
WMD (95% CI)	-2.700 (-9.438 to 4.038)	-3.824 (-6.588 to -1.060)	0.100 (-5.922 to 6.122)	-4.490 (-7.315 to -1.666)	-5.778 (-10.197 to -1.360)	-2.597 (-5.733 to 0.539)
I^2^ (%)	0	45.9	0	0	28.9	5.4
*P*	0.432	0.007	0.974	0.002	0.010	0.105
***DBP***						
No. of treatment arms	1	3	1	3	2	2
WMD (95% CI)	-1.800 (-6.348 to 2.748)	-2.695 (-7.303 to 1.912)	1.500 (-2.283 to 5.283)	-3.542 (-6.873 to -0.210)	-5.174 (-12.662 to 2.314)	-0.832 (-4.811 to 3.148)
I^2^ (%)	0	74.9	0	46.0	69.5	69.4
*P*	0.438	0.252	0.437	0.037	0.176	0.682
***WEIGHT***						
No. of treatment arms	2	2	2	2	2	2
WMD (95% CI)	-0.762 (-6.630 to 5.106)	0.087 (-4.359 to 4.532)	0.087 (-4.359 to 4.532)	-0.762 (-6.630 to 5.106)	-0.762 (-6.630 to 5.106)	0.087 (-4.359 to 4.532)
I^2^ (%)	0	0	0	0	0	0
*P*	0.799	0.969	0.969	0.799	0.799	0.969

TC, total cholesterol; LDL-c, low-density lipoprotein cholesterol; HDL-c, high-density lipoprotein cholesterol; TG, triglycerides; SBP, systolic blood pressure; DBP, diastolic blood pressure; WMD, weighed mean difference; CI, confidence intervals.

mg/dl for TC, LDL-c, HDL-c, TG; mmHg for SBP and DBP.

^2^ is inherent to I2.

### 3.7 Sensitivity Analysis and Publication Bias

There was no significant impact for any individual trial on the pooled effect sizes of meta-analyses results, so the results are reliable.

**Figure 4:**
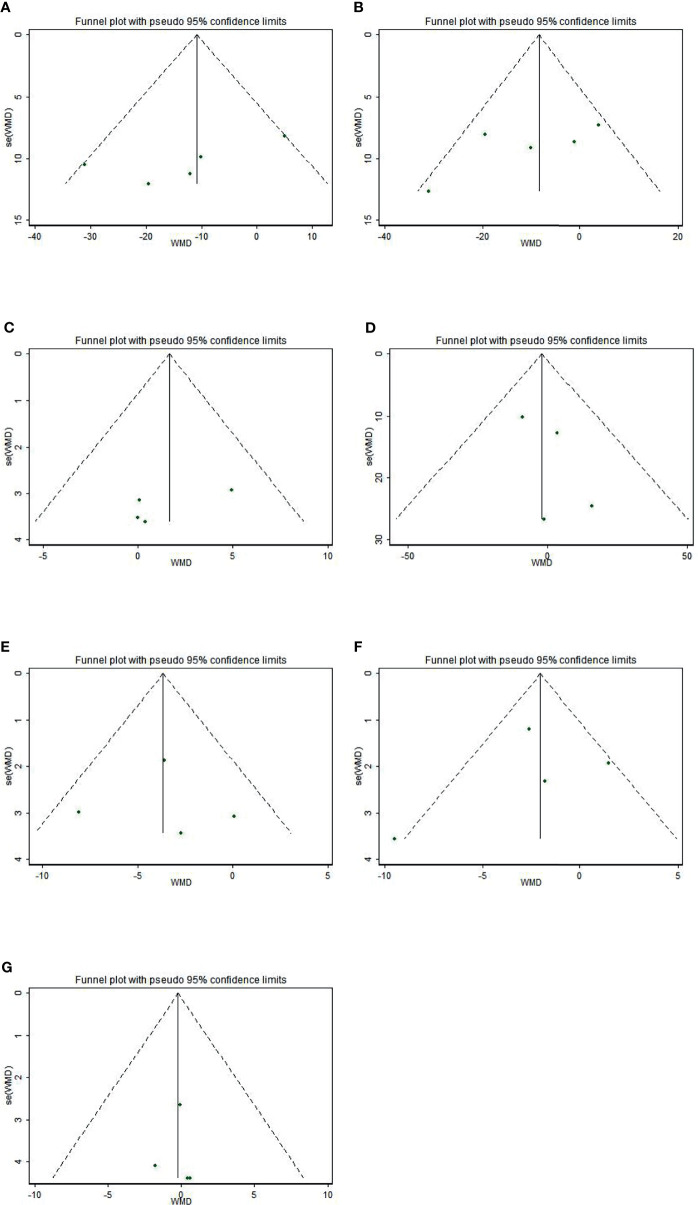
Funnel plot of the effect of sesamin supplementation on TC **(A)**, LDL-c **(B)**, HDL-c **(C)**, TG **(D)**, SBP **(E)**, DBP **(F)**, and weight **(G)**.

The funnel plots were asymmetric (**Figure 4**), indicating a possible publication bias in meta-analysis of the effects of sesamin on hemodynamics. However, the Begg’s rank correlation test and Egger’s linear regression test suggested no significant publication bias in this meta-analysis (all p>0.10) (**Table 5**).

**Table 5:** Assessment of publication bias.

	Begg’s rank correlation test			Egger’s linear regression test		
	Kendal’s score	Z value	P value	Intercept	95% CI	P value
TC	-6	-1.47	0.142	-7.052	(-17.595,3.490)	0.123
LDL	-6	-1.47	0.142	-5.415	(-15.558,4.728)	0.188
HDL	0	0	1.000	-6.138	(-21.603,9.327)	0.230
TG	2	0.68	0.497	0.956	(-2.388,4.299)	0.344
SBP	2	0.68	0.497	0.209	(-11.693,12.111)	0.947
DBP	-2	-0.68	0.497	-1.192	(-12.279,9.895)	0.689
Weight	4	1.36	0.174	-0.013	(-3.065,3.039)	0.987

## 4 Discussion

Seven articles with 212 arms were enrolled in our present meta-analysis, which assessed that sesamin supplementation did not affect the levels of HDL-c, TG, DBP, or weight, but with a decrease in TC, LDL-c, and SBP. These changes varied substantially depending on the duration, study design, and health status. In a previous meta-analysis on dietary lignans, sesamin was mentioned, but which was not analyzed alone for an accurate result (31), as we did.

Compared to the current drug therapy, dietary supplements taking sesame for an example may potentially provide a rather safe, healthy, and low-cost way to prevent disease. A previous meta-analysis by Khalesi et al. showed that sesame affected the level of TG markedly (32), and its public health implication is bright. Sesame was widely applied for its heart protection (33) which may be because of its lignans such as sesamin and sesamolin (34, 35). Sesamin is composed of carbon, hydrogen, and oxygen, whose molecular formula is C_20_H_18_O_6_, and its weight per mole is 354.35 g (36). Due to its health benefits, more and more animal and human experiments have been conducted in the past two decades.

### 4.1 Effects on Obesity

The rapid increase in obesity rates among adolescents and children around the world has shocked us (37). The status of obesity is not hopeful either, and the rise in adult obesity continues to rise (38). Since the outbreak of COVID-19, the interplay of obesity and COVID-19 has had devastating consequences with increased morbidity and mortality (39). Neidich et al. demonstrated that obese individuals were twice as likely to get the flu as healthy people who received the same vaccine (40). The health-related burden associated with obesity is estimated to have a substantial economic impact (41). Helli et al. have proved that intake of sesamin had a decrease in body weight (p = 0.001) (30). Sesamin increases lipolytic enzyme activity and decreases the activity of lipogenic enzymes, which also affects as an antagonist to liver X receptor (LXRα) and pregnane X receptor (PXR) ameliorating drug-induced hepatic lipogenesis (42, 43). However, our meta-analysis showed that the supplementation of sesamin had no association with reduction in body weight, so the effect of sesamin on weight could not be fully determined.

### 4.2 Effects on Blood Pressure

In the recent decade, hypertension is widely recognized to sharply increase the incidence of cardiovascular disease, which has dramatically increased the medical expenditure for patients around the world (44, 45). Through many *in vitro* and *in vivo* experiments, we now know that long-term effective antihypertensive therapy can avert hypertension-related mortality by nearly 50% (46). However, a systematic review written by Tadesse indicated that 45% of the subjects did not adhere to their antihypertensive medication, existing a low compliance (47). As dietary therapy becomes more and more accepted, sesamin has a good compliance prospect.

Our study revealed that a 4-week administration of 60 mg sesamin caused a decrease in BP with an average of 3.5 mmHg for SBP and 1.9 mmHg for DBP. Wu et al. proceeded a 4-week administration of sesamin and achieved a good antihypertensive effect that can greatly decrease SBP and DBP in mild-hypertensive participants (15). It was reported that patients with RA who consumed 200 mg/day sesamin showed a lower SBP level, but with no remarkable effect on DBP (30). According to Nakano (48), the mechanism of sesamin against high pressure is improving impaired endothelium-dependent vasodilatory responses. Our meta-analysis which covered both SBP and DBP levels indicated significant hypotensive effects of sesamin supplements, depending on study design, duration of treatment, and participants’ health status. Our subgroup analyses indicated the antihypertensive function of sesamin in the experimental design type was parallel. What is more, patients who received shorter than 42 days of sesamin and had an unhealthy status can show more obvious effects. Kong et al. showed that sesamin inhibited the progress of eNOS uncoupling, coupled with the effect on p-eNOS increases NO biosynthesis to relieve hypertension. Sesamin also inhibits NADPH oxidase contributing to the suppression of hypertension in hypertensive rats (49).

### 4.3 Effects on Lipid Profiles

Hyperlipidemia, a life-threatening health condition endangering the life of most patients (50), represents as increasing TC/TG/LDL-c or decreasing HDL-c (51). With elevated LDL-c concentration as a risk factor, statins can effectively reduce the concentration to play an anti-hyperlipidemia role (52, 53). Although statins’ primary application is to lower cholesterol (54), drug toxicity to the human body is still unavoidable. “Pharmacograde nutrients” may be a potentially safe, healthy, and cheap way to optimize lipid levels (55). Hirata et al. suggested that sesamin together with vitamin E can reduce the LDL-c level effectively (p<0.05) (21). Another research conducted by Mohammadshahi (22) declared that by supplementing a daily dose of 200 mg sesamin to patients with Type II diabetes, the level of TG, TC, and LDL-c became reduced. There is a biological basis of lipid lowering; Tai et al. (43)showed that sesamin might improve blood lipid by reducing hepatic steatosis and absorption of cholesterol through the intestine. Majdalawieh et al. (36) revealed that sesamin mainly played anti-hyperlipidemic roles by aiming at Δ5 desaturase, HMGCR, ABCA1, and ABCG1 through PPARα, PPARγ, LXRα, and SREBP signaling pathways, which is important for the future supplement of sesamin.

Our meta-analysis showed that daily intake of sesamin was effective in improving LDL-c and TC levels but had no significant influence on TG and HDL-c. Long-term (≥42 days) sesamin intake is more effective than a short period of supplement. The reason may be that sesamin, as a food of nutrition, can make its accumulation in the body more obvious after long-term ingestion. The meta-regression showed LDL-c’s trend becoming flat over time. Perhaps this is because LDL-c fluctuates in a dynamic range for different individuals, flattening out as its concentration nears a critical level. In the experiment conducted by Wu et al. (23), the duration was so short that sesamin played no role. Therefore, no significant influence on the indexes of lipid profile was indicated. Probably due to one crossover RCT with short-term included, findings also indicated that TC and LDL-c levels were decreased when the RCT was designed to be parallel-controlled.

Integrated data from numerous selected studies are the major superiority of our study, so the reliability and accuracy of our analysis are robust. The seven indicators we included were carefully selected and of great clinical guiding significance. What is more, the studies we included from various geographic regions around the world, so our conclusion has a wide range of application value for people in different cultural backgrounds. Additionally, our review not only provides a new food therapy target for people who are in an unhealthy status but also facilitates guidance for the development of dietary intervention therapy in the future.

The limitations of our study were self-explanatory. Firstly, a relatively small sample size of the included studies may cause a higher risk for publication bias whereas Begg’s and Egger’s tests suggested no significant publication bias in our meta-analyses. Secondly, the heterogeneity of LDL-c was relatively high, but we found that this may be caused by long duration, unhealthy status, or parallel-design trials. Thirdly, the gender ratio in the studies we included was different, and the influence of hormone level on obesity, lipid profile, and blood pressure could not be ignored.

## 5 Conclusion

Taken together, our results indicate that sesamin can be used as an easily obtainable dietary supplement to improve BP and blood lipids, and further as a health product to prevent cardiovascular diseases. In the future, large multinational prospective randomized controlled trials should help determine the ideal dose, duration, and formulation of the sesamin intervention specific to each individual patient’s health status. More multi-geographical trials with sufficient subjects, involving more countries, hoped to be conducted for the ideal dose, for the appropriate duration, and for individual patients’ fitting target.

In terms of implications for practice, the evidence from our meta-analysis suggests that unhealthy subjects taking sesamin may improve the lipid profile and BP more remarkably.

## Data Availability Statement

The original contributions presented in the study are included in the article/**Supplementary Material**. Further inquiries can be directed to the corresponding author.

## Author Contributions

Substantial contributions to the conception and design of the meta-analysis were done by JYR and YXM. The literature search and data extraction were done by YTS, JYR, and SQZ. The data analyses were done by ZAZ, ZHG, JQA, and BWY. The manuscript and revision were done by YTS, JYR, and YXM. All authors contributed to the article and approved the submitted version.

## Funding

This study was supported by the National Natural Science Foundation of China [No. 81874264].

## Conflict of Interest

The authors declare that the research was conducted in the absence of any commercial or financial relationships that could be construed as a potential conflict of interest.

## Publisher’s Note

All claims expressed in this article are solely those of the authors and do not necessarily represent those of their affiliated organizations, or those of the publisher, the editors and the reviewers. Any product that may be evaluated in this article, or claim that may be made by its manufacturer, is not guaranteed or endorsed by the publisher.

## Abbreviations

CI, confidence interval; TC, total cholesterol; TG, triglyceride; HDL-c, high-density lipoprotein-cholesterol; LDL-c, low-density lipoprotein-cholesterol; RCT, randomized controlled trial; SD, standard deviations; SE, standard error; WMD, weighted mean difference; CVD, cardiovascular diseases; PRISMA, Preferred Reporting Items for Systematic Reviews and Meta-Analysis.
