# Supplementary material for: The Effects of Sesamin Supplementation on Obesity, Blood Pressure, and Lipid Profile: A Systematic Review and Meta-Analysis of Randomized Controlled Trials
Source: Front Endocrinol (Lausanne). 2022 Mar 4;13:842152. doi: 10.3389/fendo.2022.842152 (PMC8931661; doi:10.3389/fendo.2022.842152)
Supplement: Supplementary file 1 [file DataSheet_1.docx]

**The Effects of Sesamin Supplementation on Obesity, Blood Pressure and Lipid Profile：A Systematic Review and Meta-analysis of Randomized Controlled Trials**

**Database**：*Web of Science、PubMed、Scopus、the Cochrane Library、Embase*

**Search strategy**

***Web of science 128***

TS= (sesame OR sesamin OR sesamum) AND TS= (Cholesterol OR total cholesterol OR high-density lipoprotein OR low-density lipoprotein OR triglycerides OR triacylglycerol OR blood lipids OR high-density lipoprotein cholesterol OR low-density lipoprotein cholesterol OR Lipoproteins, HDL OR Lipoproteins, LDL OR Cholesterol, HDL OR Cholesterol, LDL OR Cholesterol, VLDL OR BMI OR body weight OR blood pressure OR BP OR MAP OR PP OR pulse pressure OR diastolic blood pressure OR SBP OR systolic blood pressure) AND TS= (clinical trials OR randomized controlled trials OR controlled clinical trials OR double blind method OR single blind method OR intervention studies OR cross-over studies OR placebo OR assignment OR random allocation)

***PubMed 43***

#1 (sesame [MeSH Terms]) OR (sesamum [MeSH Terms])

#2 (sesame [Title/Abstract]) OR (sesamin [Title/Abstract]) OR (sesamum [Title/Abstract])

#3 (blood pressure [MeSH Terms]) OR (hepertension [MeSH Terms]) OR (prehepertension[MeSH Terms])

#4(blood pressure [Title/Abstract]) OR (BP [Title/Abstract]) OR (MAP [Title/Abstract]) OR (PP [Title/Abstract]) OR (pulse pressure [Title/Abstract]) OR (diastolic blood pressure [Title/Abstract]) OR (SBP [Title/Abstract]) OR (systolic blood pressure [Title/Abstract]) OR (hepertension [Title/Abstract]) OR (prehepertension [Title/Abstract])

#5 (cholesterol [MeSH Terms]) OR (total cholesterol [MeSH Terms]) OR (Lipoproteins, HDL [MeSH Terms]) OR (Lipoproteins, LDL [MeSH Terms]) OR (triglycerides [MeSH Terms]) OR (triacylglycerol [MeSH Terms]) OR (blood lipids [MeSH Terms]) OR (Cholesterol, HDL [MeSH Terms]) OR (Cholesterol, LDL [MeSH Terms]) OR (Cholesterol, VLDL [MeSH Terms])

#6 (cholesterol[Title/Abstract]) OR (total cholesterol[Title/Abstract]) OR (high-density lipoprotein[Title/Abstract]) OR (low-density lipoprotein[Title/Abstract]) OR (triglycerides[Title/Abstract]) OR (triacylglycerol[Title/Abstract]) OR (blood lipids[Title/Abstract]) OR (high-density lipoprotein cholesterol[Title/Abstract]) OR (low-density lipoprotein cholesterol[Title/Abstract]) OR (Lipoproteins, HDL [Title/Abstract]) OR (Lipoproteins, LDL [Title/Abstract]) OR (Cholesterol, HDL[Title/Abstract]) OR (Cholesterol, LDL [Title/Abstract]) OR (Cholesterol, VLDL[Title/Abstract])

#7 (body weight [MeSH Terms])

#8 (BMI [Title/Abstract]) OR (BW [Title/Abstract]) OR (body weight [Title/Abstract])

#9(Clinical Trial [ptyp]) OR (Randomized Controlled Trial [ptyp]) OR (Controlled Clinical Trial [ptyp]) (#1 OR #2) AND (#3 OR #4 OR #5 OR #6 OR #7 OR #8) AND (#9)

***Scopus 54***

( TITLE-ABS-KEY ( sesame  OR  sesamin  OR  sesamum )  AND  ALL ( cholesterol  OR  total  AND cholesterol  OR  high-density  AND lipoprotein  OR  low-density  AND lipoprotein  OR  triglycerides  OR  triacylglycerol  OR  blood  AND lipids  OR  high-density  AND lipoprotein  AND cholesterol  OR  low-density  AND lipoprotein  AND cholesterol  OR  lipoproteins,  AND hdl  OR  lipoproteins,  AND ldl  OR  cholesterol,  AND hdl  OR  cholesterol,  AND ldl  OR  cholesterol,  AND vldl  OR  bmi  OR  body  AND weight  OR  blood  AND pressure  OR  bp  OR  map  OR  pp  OR  pulse  AND pressure  OR  diastolic  AND blood  AND pressure  OR  sbp  OR  systolic  AND blood  AND pressure ) )

***the Cochrane Library 102***

#1 (sesame):ti,ab,kw OR (sesamin):ti,ab,kw OR (sesamum):ti,ab,kw

#2 (blood pressure):ti,ab,kw OR (BP):ti,ab,kw OR (MAP):ti,ab,kw OR (PP):ti,ab,kw OR (pulse pressure):ti,ab,kw OR (diastolic blood pressure):ti,ab,kw OR (systolic blood pressure):ti,ab,kw OR (DBP):ti,ab,kw OR (SBP):ti,ab,kw

#3 (cholesterol):ti,ab,kw OR (total cholesterol):ti,ab,kw OR (high-density lipoprotein):ti,ab,kw OR (low-density lipoprotein):ti,ab,kw OR (triglycerides ):ti,ab,kw OR (triacylglycerol):ti,ab,kw OR (blood lipids):ti,ab,kw OR (high-density lipoprotein cholesterol):ti,ab,kw OR (low-density lipoprotein cholesterol):ti,ab,kw OR (Lipoproteins, HDL):ti,ab,kw OR (Lipoproteins, LDL):ti,ab,kw OR (Cholesterol, HDL):ti,ab,kw OR (Cholesterol, LDL):ti,ab,kw OR (Cholesterol, VLDL ):ti,ab,kw

#4 (BMI):ti,ab,kw OR (bady weight):ti,ab,kw

#5: #1 AND (#2 OR #3 OR #4)

***Embase 200***

(sesame:ab,ti OR sesamin:ab,ti OR sesamum:ab,ti) AND (cholesterol:ab,ti OR 'total cholesterol':ab,ti OR 'high-density lipoprotein':ab,ti OR 'low-density lipoprotein':ab,ti OR triglycerides:ab,ti OR triacylglycerol:ab,ti OR 'blood lipids':ab,ti OR 'high-density lipoprotein cholesterol':ab,ti OR 'low-density lipoprotein cholesterol':ab,ti OR 'lipoproteins, hdl':ab,ti OR 'lipoproteins, ldl':ab,ti OR 'cholesterol, hdl':ab,ti OR 'cholesterol, ldl':ab,ti OR 'cholesterol, vldl':ab,ti OR bmi:ab,ti OR 'body weight':ab,ti OR 'blood pressure':ab,ti OR bp:ab,ti OR map:ab,ti OR pp:ab,ti OR 'pulse pressure':ab,ti OR 'diastolic blood pressure':ab,ti OR sbp:ab,ti OR 'systolic blood pressure':ab,ti) AND AND ('case report'/de OR 'clinical article'/de OR 'clinical trial'/de OR 'clinical trial topic'/de OR 'comparative study'/de OR 'controlled clinical trial'/de OR 'cross sectional study'/de OR 'crossover procedure'/de OR 'double blind procedure'/de OR 'experimental study'/de OR 'human'/de OR 'human experiment'/de OR 'in vitro study'/de OR 'in vivo study'/de OR 'major clinical study'/de OR 'normal human'/de OR 'parallel design'/de OR 'preclinical study'/de OR 'randomized controlled trial'/de OR 'randomized controlled trial topic'/de)

The checklist includes the following 12 items:

1. Identify the report as a systematic review

Yes

2. Provide an explicit statement of the main objective(s) or question(s) the review Addresses

Yes

1. Specify the inclusion and exclusion criteria for the review

Yes

4. Specify the information sources (such as databases, registers) used to identify

studies and the date when each was last searched

Yes

1. Specify the methods used to assess risk of bias in the included studies

Yes

1. Specify the methods used to present and synthesise results

Yes

7. Give the total number of included studies and participants and summarise

relevant characteristics of studies

Yes

8. Present results for main outcomes, preferably indicating the number of included

studies and participants for each. If meta-analysis was done, report the summary

estimate and confidence/credible interval. If comparing groups, indicate the

direction of the effect (that is, which group is favoured)

Yes

9. Provide a brief summary of the limitations of the evidence included in the review

(such as study risk of bias, inconsistency, and imprecision)

Yes

1. Provide a general interpretation of the results and important implications

Yes

1. Specify the primary source of funding for the review

Yes

1. Provide the register name and registration number

Yes
